# Characteristics and Outcomes of Generalized Pustular Psoriasis Patients Managed Through Inpatient Asynchronous Teledermatology: A Retrospective Study

**DOI:** 10.1089/tmr.2024.0068

**Published:** 2025-01-16

**Authors:** Jeffrey Chen, Joseph C. English

**Affiliations:** ^1^ University of Pittsburgh School of Medicine, Pittsburgh, PA, USA.; ^2^ Department of Dermatology, University of Pittsburgh, Pittsburgh, PA, USA.

**Keywords:** cyclosporine, generalized pustular psoriasis, infliximab, outcomes, psoriasis, teledermatology

## Abstract

**Background:**

Inpatient teledermatology (ITD) is a growing telemedicine modality aimed at addressing the shortage of dermatologists in hospitals, particularly for the management of complex skin disorders. Generalized pustular psoriasis (GPP) is a rare and potentially life-threatening condition characterized by widespread sterile pustules and systemic inflammation. Despite advances in targeted therapies, treatment is often limited to traditional agents such as cyclosporine and infliximab, due to restrictions in formulary access and insurance coverage.

**Objective:**

This study aimed to characterize the clinical features, treatment outcomes, and the impact of ITD on the management of hospitalized GPP patients at the University of Pittsburgh Medical Center (UPMC), with a particular focus on hospitalization duration and time to pustule resolution.

**Methods:**

A retrospective analysis of 35 patients with pustular skin disorders between January 2015 and August 2024 was conducted. Nine biopsy-confirmed GPP cases requiring hospitalization were included. Data on demographics, comorbidities, treatment regimens, and outcomes were collected. ITD consultations provided recommendations for corticosteroid tapering and immunosuppressive therapy initiation.

**Results:**

The cohort included nine patients, predominantly White (88.9%), with a median age of 66 years. Preexisting psoriasis and obesity were the most common comorbidities (55.6%). Leukocytosis was the most frequent lab abnormality (77.8%). The average hospitalization duration was 6.1 ± 3.8 days, and readmission occurred in 33.3% of cases. ITD consultations led to early tapering of systemic corticosteroids and initiation of immunosuppressive therapy (55.6% with cyclosporine and 33.3% with infliximab). Significant pustule improvement was achieved in 16.1 ± 7.5 days, with full resolution in 22.5 ± 17.7 days.

**Conclusion:**

ITD significantly reduced hospitalization duration for GPP patients compared with historical controls, likely due to timely therapeutic interventions. While newer biological therapies remain restricted in many hospitals, ITD facilitated the effective use of traditional immunosuppressive therapies, improving patient outcomes. This study supports the integration of ITD in hospital care models, especially in institutions lacking in-house dermatologists. Further research should explore long-term outcomes and the role of ITD in managing other emergent dermatologic conditions.

## Introduction

Inpatient teledermatology (ITD) is a burgeoning form of telemedicine that enables the remote electronic consultation of skin disorders in the hospital setting.^1^ It addresses the significant shortage of dermatologists in the United States, particularly in hospitals where dermatologic expertise may not be readily available.^2–4^ ITD has been shown to improve care of complex skin conditions by reducing misdiagnoses, unnecessary treatments, and inappropriate systemic therapies, while also lowering healthcare costs.

Generalized pustular psoriasis (GPP) is a rare but potentially life-threatening variant of psoriasis characterized by widespread sterile pustules and systemic inflammation. GPP has an estimated prevalence in the general population between 1.76 and 123 cases per million people and carries a high mortality rate, particularly in hospitalized patients, with up to 4.2% mortality due to complications, such as sepsis and cardiovascular events.^5–7^ Recent advances have implicated the IL-36 pathway in GPP pathogenesis, which has led to the development of therapies targeting this pathway, including the IL-36 receptor antagonist spesolimab.^6,7^ Despite these advancements, most patients still rely on traditional treatments, such as cyclosporine and infliximab, especially when access to newer therapies is limited by hospital formularies and insurance authorization.^6,8,9^

Studies indicate that 36.8% to 52.6% of U.S. patients with GPP are hospitalized during their initial presentation.^10,11^ Hospitalized GPP patients often require prolonged stays, typically ranging from 10 to 20 days, due to the severity of the disease and the variability in treatment pathways.^5,9,12^ Recent claims-based studies highlight the lack of standardized treatment sequences for GPP, contributing to inconsistent patient outcomes and extended hospitalizations.^8^ ITD offers a potential solution to these challenges by providing real-time expert guidance, optimizing treatment protocols, and reducing hospitalization durations.

## Objective

The objective of this study was to characterize the clinical features, treatment regimens, and outcomes of GPP patients managed through ITD at the University of Pittsburgh Medical Center. The impact of ITD on hospitalization duration and time to pustule resolution was also assessed.

## Methods

A retrospective analysis was conducted on 35 patients diagnosed with pustular skin disorders who were managed via ITD between January 2015 and August 2024. Clinical history and photographs were collected by trained health care providers and shared through the electronic medical record (EMR) system on EPIC for dermatology review. All asynchronous consultations were evaluated by the same UPMC dermatologist, with consults being completed within an average of 1.33 ± 1.76 days after admission. Dermatology recommendations often included additional laboratory tests and skin biopsies. Patient demographics, medical history, laboratory results, and pathology findings were reviewed, and final management recommendations were documented directly in the EMR.

From the initial cohort, 23 cases were diagnosed as acute generalized exanthematous pustulosis, two as palmoplantar pustular psoriasis, and one as non-biopsy-proven GPP; these were excluded from the study. The final analysis included nine biopsy-confirmed cases of GPP that required hospitalization. Of these, eight patients were seen prior to the FDA approval of spesolimab for GPP in September 2022.

All patients initially received systemic and topical corticosteroids, with subsequent adjustments made based on ITD recommendations. The consultations guided corticosteroid tapering and initiation of immunosuppressive therapies, including cyclosporine and infliximab. Primary outcomes assessed were the duration of hospitalization and time to pustule resolution.

## Results

The cohort consisted of nine patients (44.4% male, 55.6% female), and a median age of 66 years (range, 18–73 years). The majority of patients (88.9%) were White, and 66.7% had Medicare as their primary insurance. The most common comorbidities were preexisting psoriasis (55.6%) and obesity (55.6%). Cardiovascular disease and musculoskeletal disease were present in 44.4% of patients each. Leukocytosis was the most frequent lab abnormality, observed in 77.8% of patients. In 5 patients, GPP was precipitated by drugs (prednisone, amoxicillin/clavulanate, ceftriaxone, hydroxychloroquine, and dexamethasone).

In terms of disease characteristics, 77.8% of patients experienced an acute onset of pustules (defined as <2 weeks), while 22.2% had a subacute onset (>2 weeks). The average hospitalization duration was 6.1 ± 3.8 days. Readmission for GPP occurred in 33.3% of patients.

Regarding treatment, 66.7% of patients received topical corticosteroids, and 44.4% received systemic corticosteroids from the hospitalist at the time of admission. Following ITD consultations, all patients were tapered off systemic corticosteroids. Immunosuppressive therapies were initiated in 55.6% of patients with cyclosporine, 33.3% with infliximab, and 11.1% with acitretin. One patient with an indeterminate QuantiFERON-TB gold assay received cyclosporine instead of infliximab. The time to significant pustule improvement was 16.1 ± 7.5 days in 77.8% of patients, and full resolution was achieved in 22.2% of patients within 22.5 ± 17.7 days.

## Discussion

The findings of this study indicate that ITD can reduce the length of hospital stays for GPP patients. The average hospitalization duration of 6.1 days (*p* < 0.01) in this cohort is notably shorter than the 10–20 days typically reported in the literature for GPP patients managed without ITD.^5,9,10^ This reduction in hospital stay duration is likely due to the timely therapeutic interventions made possible by ITD, such as rapid tapering of corticosteroids and initiation of immunosuppressive therapies. Previous studies have shown that ITD can prevent delays in treatment and reduce unnecessary therapies, which can contribute to shorter hospitalization times.^1^

The time to pustule resolution observed in this study (16.1 ± 7.5 days for significant improvement and 22.5 ± 17.7 days for full resolution) is consistent with findings from previous studies where early intervention with appropriate therapies led to faster clinical improvement.^5,7^ ITD facilitated the early initiation of immunosuppressive therapies, including cyclosporine and infliximab, both of which have proven efficacy in controlling GPP flares. This study highlights the importance of expert-led, real-time treatment adjustments in achieving faster disease resolution.

The readmission rate of 33.3% in this cohort underscores the chronic and recurrent nature of GPP, as patients often require ongoing treatment and monitoring. However, by streamlining care with ITD, it is possible to reduce the frequency of readmissions by ensuring early and effective treatment interventions during initial hospitalizations.

The economic burden of GPP has been well documented, with prolonged hospital stays and frequent readmissions contributing to high health care costs.^12^ The use of ITD has the potential to alleviate some of these costs by reducing hospital length of stay and preventing the use of unnecessary therapies.^1^ ITD also provides hospitals without in-house dermatologists with access to expert dermatologic care, enabling consistent treatment protocols and improved outcomes for complex cases, such as GPP.

Although newer biological therapies targeting the IL-36 pathway, such as spesolimab, are promising in the treatment of GPP. However, they are not always readily accessible or restricted to outpatient use, as in our health system. As this study demonstrates, traditional therapies such as cyclosporine and infliximab remain effective when used in conjunction with ITD to ensure timely intervention. Future studies should explore the long-term outcomes of patients managed with ITD and examine its role in broader dermatologic conditions.

## Conclusion

ITD is an effective tool for managing generalized pustular psoriasis in hospital settings. ITD facilitates timely treatment adjustments, reduces hospitalization durations, and accelerates the resolution of pustules. This study supports the continued integration of ITD into hospital care models, especially in institutions without dermatology departments. Limitations in our study include a small sample size and a lack of inpatient GPP controls managed by dermatology hospitalists. Further research is needed to explore the long-term outcomes of ITD in GPP management and its potential for other emergent skin conditions.

## IRB Approval Status

Reviewed and approved by University of Pittsburgh School of Medicine.

## Abbreviations Used

EMR: electronic medical record
GPP: generalized pustular psoriasis
ITD: inpatient teledermatology
UPMC: University of Pittsburgh Medical Center
